# Clinical Evaluation of a Mobile Heart Rhythm Telemonitoring System

**DOI:** 10.5402/2012/192670

**Published:** 2012-10-14

**Authors:** Hristo Mateev, Iana Simova, Tzvetana Katova, Nikolay Dimitrov

**Affiliations:** ^1^Clinic of Cardiology, National Cardiology Hospital, 1309 Sofia, Bulgaria; ^2^Department of Noninvasive Cardiovascular Imaging and Functional Diagnostics, National Cardiology Hospital, 65 Koniovitsa Street, 1309 Sofia, Bulgaria; ^3^Department of Cardiology, University Hospital “St. Ekaterina”, 1431 Sofia, Bulgaria

## Abstract

*Purpose*. To evaluate the clinical applicability of a telemonitoring system: telemetric system for collection and distant surveillance of medical information (TEMEO). *Methods*. We evaluated 60 patients, applying simultaneously standard Holter ECG and telemonitoring. Two different comparisons were performed: (1) TEMEO ECG with standard 12-lead ECG; (2) TEMEO Holter with standard ECG Holter. *Results*. We found a very high coincidence rate (99.3%) between TEMEO derived ECGs and standard ECGs. Intraclass correlation coefficient analysis revealed high and significant correlation coefficients regarding average, maximal, and minimal heart rate, % of time in tachycardia, single supraventricular ectopic beats (SVEB), and single and couplets of ventricular ectopic beats (VEB) between Holter ECG and TEMEO derived parameters. Couplets of SVEB were recorded as different by the two monitoring systems, however, with a borderline statistical significance. *Conclusions*. TEMEO derived ECGs have a very high coincidence rate with standard ECGs. TEMEO patient monitoring provides results that are similar to those derived from a standard Holter ECG.

## 1. Introduction

Telemedicine is a relatively new medical trend which incorporates medicine, telecommunications, and information technologies, providing diagnostic workup, treatment, consulting, and training. It enables a patient to get specialized medical advice 24 hours a day independent of his/her location. Telemedicine has been acknowledged in world-leading countries and there are a large number of clinical trials and even some medical journals devoted entirely to this topic. Most of telemonitoring studies however focus on heart failure population [1–5], about 3500 patients included in total, with different kinds of data transmission. Results from most [1–4], but not all [5], of these studies have shown that telemonitoring can be effective in clinical management of patients.

Experience with ECG monitoring is still insufficient. Most of the results come from laboratory tests and small clinical trials [6–16], while data transmission via mobile network is rare.

Telemonitoring systems are designed in various ways and adapted to detect and transmit information regarding different clinical parameters. Systems that detect RR intervals and are able to record electrocardiograms (ECG) do not yet have a large clinical application. After performing a literature search we were not able to find a clinical study comparing such a system with the gold standard for ambulatory ECG monitoring—Holter ECG system.

Considering this and some existing controversies regarding the usefulness and clinical application of telemonitoring in daily clinical practice we set ourselves the task to evaluate the clinical applicability, patient compliance, and convenience when using a telemonitoring system, telemetric system for collection and distant surveillance of medical information (TEMEO). Two different parameters were compared: (1) TEMEO ECG and standard 12-lead ECG, where only rhythm, heart rate (HR), supraventricular ectopic beats (SVEB), ventricular ectopic beats (VEB), and significant pauses are compared, and (2) comparison between standard Holter ECG and TEMEO Holter regarding average, maximal, and minimal HR, total number of analyzed complexes, percentage of time in tachycardia and single, and couplets and triplets of supraventricular and ventricular ectopic beats.

## 2. Methods

### 2.1. Patient Group and Study Design

The study was conducted between March and October 2010. We included 60 in-hospital patients, selected from the daily list of patients hospitalized in cardiology departments in our institution. In our opinion, the planned number of patients is sufficient to investigate the functionality and reliability of the system in this pilot study. Inclusion criteria were: every 5th hospitalized patient in a cardiology department, age interval 35–79 years, and presence of cardiovascular disease: coronary artery disease, rhythm-conduction disturbances, congenital or acquired valvular heart disease, and so forth. Exclusion criteria were: implanted pacemaker, resynchronization device or cardioverter-defibrillator; a physical or emotional condition that, according to the investigator, will impede patient participation in the study (e.g., in critically ill patients or those in intensive care units); thoracic deformities and lack of informed consent.

The design of the study included simultaneous Holter ECG and TEMEO monitoring for at least 20 hours. The protocol of the study included also 5 simultaneous ECG recordings (TEMEO and standard ECG) at baseline, and afterwards at 1st, 2nd, 6th hour and at the end of the monitoring period. All of the manipulations, recordings, and interpretation were performed by the study investigators.

Some medical imaging procedures like X-ray examination and echocardiography are performed before using TEMEO system (in terms of easier access to the chest in case of echocardiography).

After evaluation of inclusion and exclusion criteria and signing the informed consent form each patient was briefly instructed on how to wear and manipulate (recording ECG) with TEMEO system. The device has only two buttons—one with ON/OFF function and one for ECG record. ECG recording with TEMEO is very easy—stable positioning of the device on the chest, pushing ECG record button for 3 sec, and waiting in this position for 10 seconds. The device was installed and after it a Holter ECG monitor was also added.

After completion patients' opinion of the telemonitoring system was registered, as well as any possible reported adverse events.

### 2.2. TEMEO System

TEMEO system was designed and developed two years ago, as a true wireless telemonitoring solution. The first step was development of its 24 hour ECG Holter-like capability, mobile network transmission, and software for automatic ECG data analysis. The second step was the development of one lead ECG record capability. The next step, which is under development, is blood pressure monitoring capability.

TEMEO is an ambulatory patient telemonitoring system, which for the purpose of this study was tested in hospital surroundings.

The general conception of TEMEO telemonitoring system is not for it to be a complex and complicated system but to be simple, friendly, mobile, and easy to use. As it is intended to be used by outpatients for a long period of time (weeks), without any medical supervision, it is designed to operate with only short training of patients. Most of the components are designed according to these requirements.

**Figure 1:**
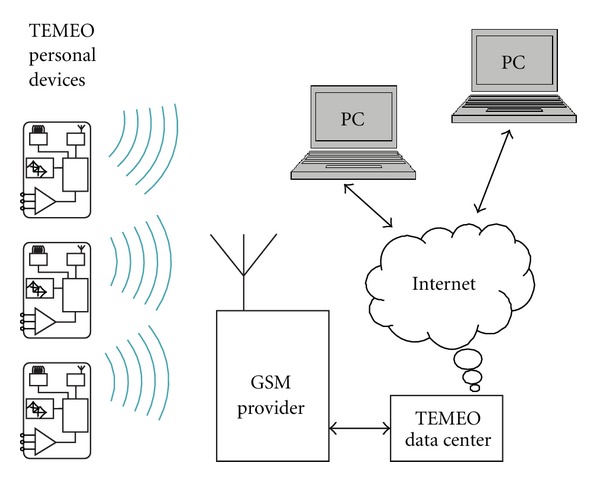
TEMEO heart rate monitoring system—principles of work.

The system consists of an elastic chest belt, handheld TEMEO device, and TEMEO electronic center with GSM connection between the device, and the electronic center (Figure 1).

#### 2.2.1. TEMEO Belt (Figure 2)

An elastic belt placed on the chest under the breasts registers precordial electrical activity of the heart and detects R-R intervals. Two detecting electrodes are built in on the inner surface of the belt, in close contact with the skin. Data from the belt is registered continuously, and is transmitted wirelessly to the mobile handheld device.

TEMEO belt could be a source of noise, but is more comfortable, easier to place and remove, and skin friendlier than sticky (standard Holter) electrodes for a long period of time.

#### 2.2.2. TEMEO Handheld Device (Figure 3)

The handheld device could be worn on the belt or placed in proximity to the patient, for example, during the night period. By means of a GSM network, within 5 minute intervals, recorded data is transmitted (GPRS standard used) to the TEMEO electronic center for further analysis and visualization. GPRS standard for wireless transmission is used because data transfer rate is sufficient; GPRS is a well known and widely used standard supported by Bulgarian mobile operators.

This device, due to 3 active (+ one inactive) pin-electrodes placed on the back, has single lead recording capability. When placed vertically at the left sternal border it records lead vector potential similar to a standard aVF. ST-T change analysis is impossible due to minor position and/or angle differences between recordings. The one-lead recording capability does not allow proper diagnosis of ventricular tachycardia.

TEMEO ECGs are recorded only on demand, after proper placement and button push, and are transmitted immediately, via GPRS to the electronic center. ECG sampling rate is 100 Hz and the bandwidth used −0.5–40 Hz. Transmission rate over wireless is done with GPRS Class 12 and no compression is employed. At the present moment the system does not have the capability to record continuously the ECG signal 24 hours a day, 7 days a week.

The mobile device has also an accelerometer for detection of physical activity (and therefore differentiating between resting condition and physical exertion) simultaneously with R-R interval detection. The battery is irremovable. A new version with a removable battery is under development.

Recorded data is transmitted within 5 minute intervals—that means that every 5 min GPRS connection is established for some seconds, which is feasible, easily achieved, and cheap. The only limitation is GSM coverage, which is 96–98% of the territory of the country. Roaming data transfer is also possible, but it is more expensive. The battery provides 20–24 hours of continuous work (depending on the number of TEMEO ECG records) and 4 hours for charging are required. If there is a loss of signal, a red indicator starts blinking, alerting of the problem. At the moment of system evaluation, if collected data is not transmitted, it is lost due to continuous recording of new data. Now there is an option for 60 minute record buffering.

#### 2.2.3. Electronic Center

TEMEO electronic center consists of a server with developed software for automatic R-R interval analysis [17, 18]—for rhythm detection (including atrial fibrillation), heart rate, and premature beats.

The method for beat detection that we use in data analysis is one of the best known in the scientific literature. It has been tested with the MIT-BIH arrhythmia database (sensitivity Se = 99.74% and specificity Sp = 99.65%) [17] and with AHA database (sensitivity Se = 99.78% and specificity Sp = 99.85%) [19].

The electronic center is accessible via the internet, from any PC or smartphone with previously installed TEMEO software, with security access to the system. The telemonitoring system has the possibility to alert via SMS the monitoring doctor and the patient in case of significant deviation from normal values (e.g., heart rate less than 40 beats per minute).

### 2.3. Holter ECG Recordings

ECG Holter was performed using SYGNA-Lyser, Long Term Ambulatory ECG analysis, Signa Cor Laboratory, Version 5.2.1.3 08.04.2008. SYGNA-Lyser SD ECG Holter device has max 200 Hz sample rate and resolution 8 bit. The recordings were analyzed by an experienced study investigator, blinded for the TEMEO analysis results.

### 2.4. ECG Recordings

Standard 10-second-long 12 lead ECGs were recorded using Sicard 460, Siemens and were analyzed by an experienced study investigator, blinded for the TEMEO ECG results.

One-lead 10-second-long TEMEO ECG recordings were registered in supine position, with the handheld device placed vertically at the left sternal border, just under the clavicle (lead vector similar to an aVF), and were compared with corresponding standard ECGs. Comparison of a single-lead ECG with 12-lead ECG was performed, but only for above-mentioned parameters.

These two types of ECG recordings (TEMEO ECG and standard ECG) were performed simultaneously at baseline, on the 1st, 2nd, and 6th hour and at the end of the monitoring period.

### 2.5. Statistics

We tested the distribution of continuous variables using the Kolmogorov-Smirnov test. Normally distributed data were presented as mean ± standard deviation (SD), whereas nonnormally distributed data were presented as median and interquartile range (IQR) (the difference between the 25th and 75th percentile). Categorical variables were presented in percentage terms.

#### 2.5.1. ECG Capability Comparison

We analyzed ECG recordings considering 5 parameters: heart rhythm (sinus rhythm, atrial fibrillation, and atrial flutter), HR, SVEB, VEB, and significant pauses (>2500 ms). For these parameters we have reported the percentage of coincidence (accordance) between TEMEO and standard Holter ECG systems.

#### 2.5.2. ECG Holter Capability Comparison

Regarding the monitoring period we have evaluated the following parameters: average, maximal, and minimal HR during the recorded period, total number of analyzed complexes, percentage of time in tachycardia, and single, couplets, and triplets of SVEB and VEB. These parameters were then compared using correlation analysis and calculating the intraclass correlation coefficients. A two-tailed *P* value <0.05 was considered statistically significant. Statistical analysis was performed using SPSS statistical software for Windows, version 13.0.

### 2.6. Ethics

Patients were included in the study after signing an informed consent form. The protocol of the investigation was approved by the Local Ethical Committee. The study was in accordance with the Declaration of Helsinki.

Before inclusion we made sure that every patient was aware of the possibility to discontinue the examination without any negative consequences.

**Adverse Events**

No adverse events were reported during the study period.

## 3. Results

### 3.1. Study Group

In the present study we included 60 patients, 22 (37%) of them female, mean age—58 ± 8 y. The demographic characteristics, risk factor distribution, and medical history of the patients are presented in Table 1.

### 3.2. ECG Capability Comparison

**Figure 4:**
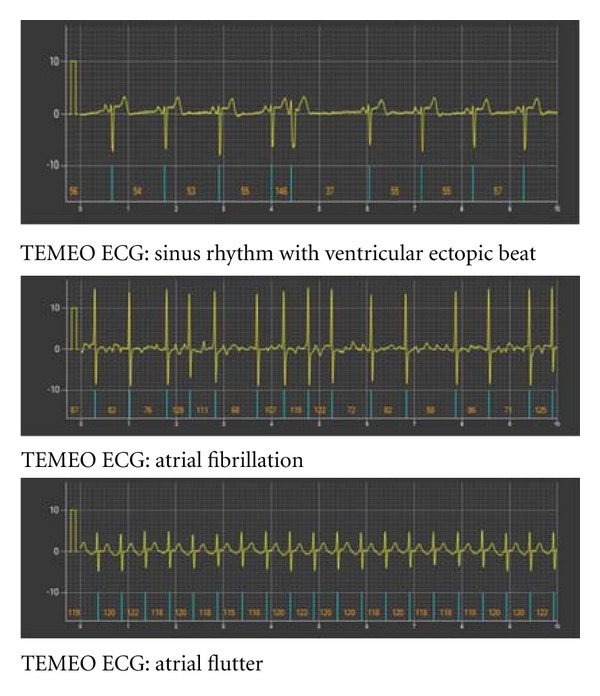
Examples of TEMEO ECG recordings.

We have performed 297 standard ECG recordings and 297 TEMEO ECGs (mean number of ECGs per patient—4.95 for each mode of recording) (Figure 4). Due to technical difficulties 3 standard and 3 TEMEO ECGs were not performed. We have performed 1485 comparisons—297 standard ECGs compared with 297 TEMEO ECGs with regard to the 5 criteria mentioned above.

We found a very high coincidence rate of 99.3% when TEMEO derived ECGs were compared with standard ECGs. We have not registered a coincidence in only 10 comparisons (0.7%). Differences in HR were found in 8 cases—a disparity of 3–6 beats per min, all in ECGs of patients with atrial fibrillation. In all cases with sinus rhythm, it is correctly interpreted in all recorded TEMEO ECG records, even though there were small differences in device position on the chest. In one case, TEMEO ECG was interpreted as atrial fibrillation, when in fact it was atrial flutter with variable AV conduction. In another case, a supraventricular ectopic beat was not registered in TEMEO ECG. In all standard ECGs with atrial fibrillation (8.7% of the recordings), the rhythm was correctly interpreted in the corresponding TEMEO ECG recordings. In 12 standard ECGs with atrial flutter, the rhythm was correctly interpreted in 6 corresponding TEMEO recordings, one of the TEMEO ECGs was interpreted as AF, and in 5 cases the interpretation was atrial tachycardia with 2 : 1 conduction.

Twenty TEMEO records had small artifacts; in all cases, however, the quality was good enough for proper determination of all examined parameters.

### 3.3. ECG Holter Capability Comparison

The overall monitoring time with TEMEO system was 61 389 minutes/1023 hours/42.6 days; with the standard Holter ECG it was 72 778 minutes/1213 hours/50.5 days. That means that TEMEO monitoring comprises 84.3% of standard Holter monitoring time. Fifteen patients (25%) were monitored <12 hours with both systems, while the rest of the patients were monitored between 12 and 25 h.

In order to compare the telemonitoring device with standard Holter ECG we have analyzed only this time period of the Holter ECG recording during which there is a simultaneous TEMEO recording.

The correlations and intraclass correlation coefficients (ICC) between average, maximal, and minimal HR during the recorded period, total number of analyzed complexes, percentage of time in tachycardia, and single and couplet SVEB and VEB evaluated with standard ECG Holter and TEMEO systems are presented in Table 2. Comparisons of triplets (SVEB and VEB) between the systems were not correct (and therefore not mentioned here) because of the relatively small number of triplets in the recordings.

As could be seen from Table 2 there was a very good coincidence between the two systems regarding average and minimal HR and % of time in tachycardia (*P* < 0.001) and statistically significant accordance in the results concerning maximal HR, single SVEB, and single and couplet VEB (*P* < 0.05). Comparison regarding couplets of SVEB between both systems showed a borderline statistical significance.

Maximal and minimal HR determined automatically with the SYGNA-Lyzer system were often interpreted after manual evaluation as erroneously detected due most probably to artifacts. That is the reason why maximal and minimal HR were determined manually by the investigator and as could be seen from Table 3 these manually determined values correlated more closely (and were statistically significant) with the maximal and minimal HR recorded during TEMEO monitoring.

There were artifacts recorded with both systems—5% of the time of the Holter ECG recording comprised of artifacts, which was not significantly different from the 6.3% duration of artifact-recording with TEMEO system.

### 3.4. Patients' Compliance

During the present study the conditions of wearing the device were not normal—two systems worn together and with multiple ECG recordings (again simultaneous) during the monitoring period. Nevertheless compliance with the system was excellent: none of the patients refused to complete the monitoring period and none of them complained about some discomfort or adverse reaction.

Generally the degree of comfort depends on the tightening of the belt: the looser the belt, the more comfortable its wearing, but also more artifact prone. If there is bad contact with the skin, short warning sound signal is present to tighten the belt. TEMEO belt does not disturb normal respiratory excursions during rest and mild and moderate physical activity (some patients with stroke use TEMEO during physical rehabilitation program).

TEMEO is not suitable for women with a very large bust due to problems with positioning of the belt. TEMEO system is not water resistant.

For the typical situation of ambulatory long-term patient monitoring we have a standard evaluation form for every patient, with 6 questions about the feasibility, comfort, and some comments for the system. Our experience is that 2–4 week monitoring with the system is easy, comfortable, does not restrict their daily activity, and patients are sufficiently motivated to perform it.

## 4. Discussion

With the development of electronics and its application in medicine it is possible to transmit and process many vital parameters of the human body. The most important, and in this moment the most interesting, signal for monitoring and analyzing is the ECG signal.

ECG telemonitoring enables the control of the health status despite the spatial separation of patient and physician. Cardiac arrhythmias, palpitations of unknown causes, the outcome of antiarrhythmic drug therapy, or interventional ablation therapy can be diagnosed using ECG telemonitoring. The use of telemonitoring for ECG signals has many advantages compared to a classical patients' ECG reading, such as: covering many patients at the same time, covering a large area, real-time information, faster diagnosis, timely therapy, and prevention.

There are two widely applicable methods for ECG data recording and analysis: a single-moment ECG capture during patient examination and a 24-hour ECG recording with a later analysis—Holter monitoring (there is also a possibility for continuous ECG monitoring in the setting of intensive care unit, applicable, however, only for critically ill patients). The disadvantage of the 1st method is that it is a single-moment registration and the information is usually not enough to make a diagnosis. The shortcoming of the 2nd method is that it is not possible to intervene immediately, which sometimes can have serious consequences. One possiblesolutionof the problem is continuous loop recorders, even implantable, with their capability for ECG recording and transfer, only during an event.

TEMEO system has a potential to combine these two methods of examination in one functional monitoring system with its options for continuous monitoring for long periods of time with multiple event ECG recording. The system is good enough to register episodes of atrial fibrillation, even short standing and “silent”. At this moment, this is the most promising application of this telemonitoring system. TEMEO system is under further investigation with outpatient patients with possible atrial fibrillation. We investigate ambulatory patients with ischemic stroke (>1 month after the acute period) of unknown reason in order to detect any cryptogenic atrial fibrillation responsible for the ischemic incident. At this moment, we have more than 20 patients monitored for a period between 22 and 32 days (mean period 26 days) during their ordinary activity at home and none of them was admitted in hospital during the monitoring period. Some of the patients performed ambulatory rehabilitation program with TEMEO monitoring and even in theses circumstances the compliance was also excellent. TEMEO monitoring time was more than 70% of monitoring period (note that during charging the system is switched off), which in our opinion is a good indicator for excellent compliance.

TEMEO system is planned to be user friendly, easy to position on the chest, appropriate for long-period monitoring (even one month). The option with 2-3 or even 12 lead ECG records, requires more electrodes and more reliable contact with the skin than is possible with the TEMEO belt, which, in our opinion, has lots of disadvantages for a long period examination. In most cases, one lead ECG record was quite enough to register most frequent rhythm disturbances.

In the present study we have evaluated 60 patients, applying simultaneously standard Holter ECG and TEMEO telemonitoring system. Patient compliance during the monitoring period was excellent. We have compared TEMEO derived ECGs with standard ECGs (279 comparisons) and have found a very high coincidence rate of 99.3%. On the basis of intraclass correlation coefficient analysis we then evaluated the level of agreement between Holter ECG derived and TEMEO derived parameters and found high and statistically significant correlation coefficients regarding average, maximal, and minimal HR (especially when the comparison was performed with the manually measured Holter ECG data), % of time in tachycardia, single SVEB, and single and couplet VEB (*P* < 0.05). Comparison regarding couplets of SVEB between both systems showed a borderline statistical significance.

According to the literature search we have performed, this is the first study comparing an HR/ECG telemonitoring system with a standard Holter ECG device. There is great research progress in detection, wireless transfer, and automatic analysis of ECG signals, but clinical experience with working telemonitoring systems is very limited.

The first trial evaluating ECG data transfer capability was the one of Zhang et al., with phone line ECG transfer [7]. With development in technology, telemonitoring systems became more sophisticated and data transfer was improved. In the last 5 years at least 10 laboratory experiments and small trials were performed with wireless data transfer [6–16]. Some of them investigated WAP data transfer [13]. ECG monitoring system was applied in different subsets of patients: high risk postmyocardial infarction patients, to detect life threatening arrhythmias [11] and healthy athletes monitored during physical activity [12]. Most of the studies implied a single-channel ECG, with the exception of the study byAmien and linwith two-channel ECG [15], and the one byVukajlovic et al. with 12 (signal reconstruction) lead detection system [10]. As the results are promising, the investigation in this area continues.

There are some limitations in the present study, namely, it was initially planned to monitor patients for at least 20 hours but due to some technical limitations (most often loss of GSM coverage) the monitoring period was shorter. Furthermore, TEMEO monitoring time was 15% shorter than standard Holter surveillance period. This we have corrected for, taking only that period from the Holter ECG recording, during which we have a simultaneous recording from the telemonitoring system.

The software of the Holter ECG system SYGNA-Lyzer did not allow us to perform some other comparisons, such as dividing the day and night period and comparing the parameters individually for these periods between the systems (TEMEO has the advantage of giving the possibility to divide the whole monitored period to an indefinite number of subperiods and providing separate analysis for each of them).

Due to minor position/angle differences of positioning ECG record device, TEMEO is not appropriate for ischaemia detection. Ventricular tachyarrhythmias could not be properly diagnosed with the system due to one-lead ECG recording capability. A 12-lead telemonitoring system is under development.

## 5. Conclusion

TEMEO telemonitoring system is convenient for the patient, and associated with an excellent compliance. TEMEO derived ECGs have a very high coincidence rate with standard ECGs. TEMEO patient monitoring provides results that are similar to those derived from a standard Holter ECG for some parameters.

Any additional data is available on http://temeo.org/.

## 6. Future Perspective

Telemedicine and telemonitoring is a rapidly evolving, contemporary, and promising field in clinical cardiology. ECG telemonitoring is bound to find wide clinical application due to the prevalence of rhythm and conduction disorders and their unpredictable appearance in the daily life of our patients.

The ability of 12-lead ECG telemonitoring (a current line of development for TEMEO) will further expand the possibilities for device's application, including ventricular tachycardia detection and ST segment (ischemia) evaluation.

## Figures and Tables

**Figure 2:**
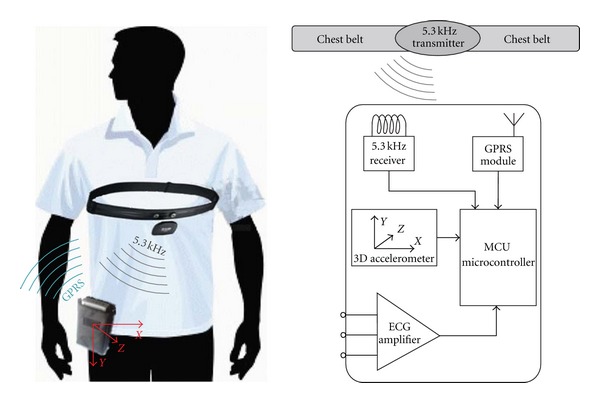
TEMEO detecting and transmitting system. Principal scheme of handheld device is seen.

**Figure 3:**
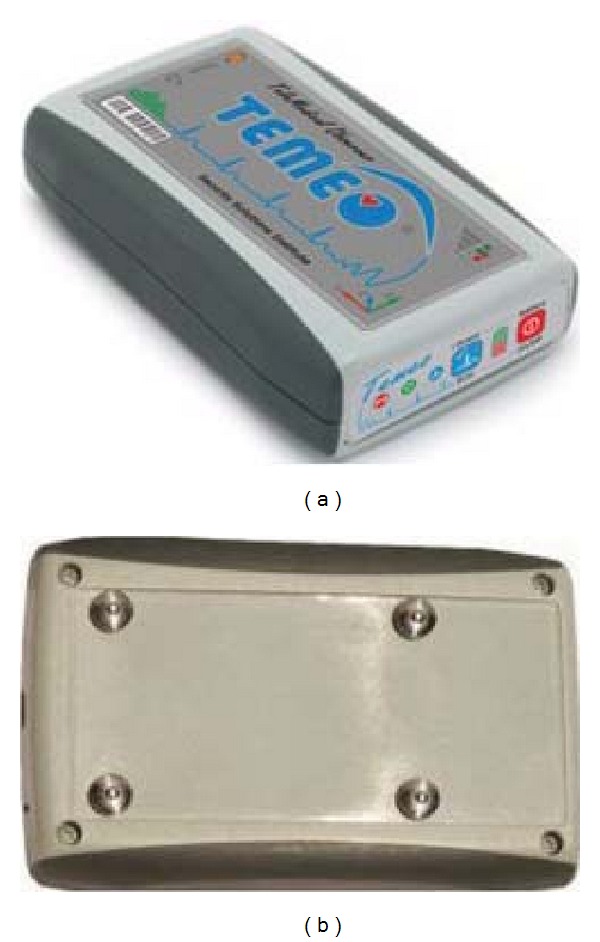
TEMEO handheld device: top and bottom view. Four pin electrodes are well seen.

**Table 1:** Demographic characteristics, risk factor distribution, and medical history of the study group.

Parameter	Distribution
Patients—(%)	60 (100%)
Gender	
Male—(%)	38 (63.3%)
Female—(%)	22 (36.7%)
Age	
Overall (mean ± SD)	58 ± 8 y
Men (mean ± SD)	56 ± 9 y
Women (mean ± SD)	60 ± 7 y
Risk factors	
Arterial hypertension—(%)	53 (88.3%)
Dyslipidemia—(%)	41 (68.3%)
Diabetes mellitus—(%)	20 (33.3%)
Smokers—(%)	20 (33.3%)
Medical history	
Stable angina pectoris—(%)	11 (18.3%)
Unstable angina pectoris—(%)	25 (41.7%)
Previous myocardial infarction—(%)	7 (11.7%)
Previous PCI—(%)	14 (23.3%)
Previous CABG—(%)	3 (5%)
Valvular disease—(%)	3 (5%)
Heart failure—(%)	2 (3.3%)
Syncope—(%)	2 (3.3%)
Permanent atrial fibrillation—(%)	3 (5%)
Paroxysmal atrial fibrillation—(%)	12 (20%)
Atrial flutter—(%)	1 (1.67%)
Paroxysmal supraventricular tachycardia—(%)	1 (1.67%)

SD: standard deviation; PCI: percutaneous coronary intervention; CABG: coronary artery bypass grafting.

**Table 2:** Correlations and intraclass correlation coefficients (ICC) between different parameters measured with standard Holter ECG and TEMEO monitoring system.

Parameter	ECG Holter	TEMEO system	Correlation	ICC	*P* value
Average HR	75 ± 14	74 ± 16	0.85	0.91	<0.001
Maximal HR	130 ± 33	121 ± 19	0.42	0.48	0.02
Minimal HR	54 ± 14	48 ± 13	0.77	0.84	<0.001
% time in tachycardia	10.2 ± 16.3%	9 ± 15.1%	0.59	0.72	<0.001
VEB single	456 ± 618	363 ± 722	0.43	0.58	0.02
VEB couplets	109 ± 258	76 ± 195	0.75	0.47	0.01
SVEB single	815 ± 1002	315 ± 678	0.68	0.59	0.001
SVEB couplets	105 ± 218	33 ± 106	0.19	0.24	0.05

HR: heart rate; VEB: ventricular ectopic beats; SVEB: supraventricular ectopic beats.

**Table 3:** Correlations and intraclass correlation coefficients (ICC) between maximal and minimal HR measured with standard Holter ECG holter, TEMEO monitoring system, and manually.

Parameter	ECG Holter	Manual	TEMEO system	Correlation	ICC	*P* value
Maximal HR	133 ± 33	113 ± 23		0.3	0.44	0.014
		113 ± 23	121 ± 19	0.68	0.8	<0.001

Minimal HR	55 ± 14	56 ± 11		0.9	0.93	<0.001
		56 ± 11	48 ± 13	0.72	0.83	<0.001

HR: heart rate.
